# Poor peer relations predict parent- and self-reported behavioral and emotional problems of adolescents with gender dysphoria: a cross-national, cross-clinic comparative analysis

**DOI:** 10.1007/s00787-015-0764-7

**Published:** 2015-09-15

**Authors:** Annelou L. C. de Vries, Thomas D. Steensma, Peggy T. Cohen-Kettenis, Doug P. VanderLaan, Kenneth J. Zucker

**Affiliations:** Center of Expertise on Gender Dysphoria, VU University Medical Center, PO Box 7057, 1007 MB Amsterdam, The Netherlands; Gender Identity Clinic, Child, Youth, and Family Services, Centre for Addiction and Mental Health, Toronto, ON Canada; Department of Psychology, University of Toronto Mississauga, Mississauga, ON Canada

**Keywords:** Gender dysphoria, Gender identity disorder, Transgender, Child Behavior Checklist, Youth Self-Report Form, Behavioral and emotional problems, Adolescents, Peer relations

## Abstract

This study is the third in a series to examine behavioral and emotional problems in children and adolescents with gender dysphoria in a comparative analysis between two clinics in Toronto, Ontario, Canada and Amsterdam, the Netherlands. In the present study, we report Child Behavior Checklist (CBCL) and Youth Self-Report (YSR) data on adolescents assessed in the Toronto clinic (*n* = 177) and the Amsterdam clinic (*n* = 139). On the CBCL and the YSR, we found that the percentage of adolescents with clinical range behavioral and emotional problems was higher when compared to the non-referred standardization samples but similar to the referred adolescents. On both the CBCL and the YSR, the Toronto adolescents had a significantly higher Total Problem score than the Amsterdam adolescents. Like our earlier studies of CBCL data of children and Teacher’s Report Form data of children and adolescents, a measure of poor peer relations was the strongest predictor of CBCL and YSR behavioral and emotional problems in gender dysphoric adolescents.

## Introduction

Adolescents with gender dysphoria (GD) present with a marked incongruence between their experienced/expressed gender and assigned gender at birth [1]. They typically have a strong desire for medical gender affirming reassignment treatment once secondary sex characteristics start to develop. The availability of puberty suppression has changed clinical management [2] of GD youth substantially and might be one of the causes of a steep increase in referrals to most specialized gender identity clinics [3], a trend that has been noted in both Europe and North America [4]. As a consequence, mental health professionals working in general mental health care settings will also be confronted more frequently with gender dysphoric adolescents.

One of the important clinical and theoretical issues that arise when assessing these adolescents is if, apart from GD, they have other types of behavioral and emotional problems and, if so, how this should be understood. Social ostracism (e.g., rejection by peers, teasing by peers, etc.) has been suggested as an important factor leading to psychological distress, which is more prevalent in gender variant or gender non-conforming youth compared to children and adolescents showing gender stereotypical behavior [5–8]. As gender dysphoric children and adolescents present at the extreme end of the gender non-conformity spectrum, as they are identifying in most respects with the other gender, it might be expected that they are particularly susceptible to peer rejection and behavioral and emotional problems.

The early literature focused predominantly on the psychological functioning of gender dysphoric children [9, 10], but more recent studies have also examined the psychological functioning of gender dysphoric adolescents. These studied have found high prevalence rates of psychiatric comorbidity as compared to what is reported in the general population. For example, in two chart review studies from clinics in the UK and the US, more than half of the referred adolescents (124 and 97 cases, respectively) suffered from depression [11, 12]. A Canadian study of 84 adolescents reported a somewhat lower rate of 35 % [13], which was similar to the percentage of youth (total *n* = 101) classified with at least mild depression on the Beck Depression Inventory in a US study [14]. Using the standardized Diagnostic Interview Schedule for Children) [15], the prevalence rate of those who had one or more other psychiatric disorders (including mood disorders) among a cohort of 105 gender-dysphoric adolescents seen in a specialized gender identity clinic in Amsterdam was 32.4 % [16].

Two large specialized gender identity clinics for children and adolescents, located in Toronto and Amsterdam, have employed standardized questionnaires to assess the behavioral and emotional problems in gender dysphoric youth, which has allowed us to conduct a systematic cross-national, cross-clinic comparative analysis. These are the Child Behavior Checklist (CBCL), the Youth Self-Report (YSR), and the Teacher’s Report Form (TRF) [17–19].

In our first study of 488 GD children (age range, 3–12 years), it was found that, on average, parent-reported problems on the CBCL were comparable to that of clinic-referred children in general and higher than non-referred children, with no significant between-clinic difference [20]. There was no significant difference in the number of behavior problems in the natal GD boys vs. girls, but both sexes had more Internalizing problems than Externalizing problems. In a second study, the TRF was used in a combined sample of 728 children and adolescents [21]. Teachers reported significantly more problem behavior in the latter group, and the natal boys had more Internalizing than Externalizing problems but the natal girls did not. In contrast to the CBCL data reported by Cohen-Kettenis et al. [20], there was evidence for a between-clinic difference, with children and adolescents in Toronto showing more behavioral and emotional problems compared to the Amsterdam sample. In both studies, a 3-item scale was constructed from the CBCL/TRF to create an index of poor peer relations [22], which proved to be the strongest predictor of other CBCL/TRF problems in multiple regression analyses.

In adolescents, self-report measures become an important source of information on their experienced emotional and behavioral problems, as correlations between other informants drop compared to children [23]. There are three studies using the YSR (one from the Toronto clinic, one from a selected sample of the Amsterdam clinic, and one from a London based clinic) that have revealed quite similar results with problem scores comparable, on average, in severity to referred youth in the standardization sample [16, 24, 25].

The present study is the third in our series of cross-national, cross-clinic comparisons between the Toronto and Amsterdam gender identity clinics, this time with an analysis of CBCL and YSR data in our adolescents. The aims of this study were three fold: (1) to characterize the patterns of behavioral and emotional problems in the two clinics by both parent-report and self-report; (2) to identify the similarities and differences in behavioral and emotional problems between the two clinics; and (3) to identify the predictors of behavioral and emotional problems using the same variables that were used in our two previous studies.

## Method

### Participants

The Dutch clinic was first established in 1987 at the University Medical Center Utrecht in Utrecht. It moved to the VU University Medical Centre (formerly named the Free University Academic Hospital) in Amsterdam in 2002. The Toronto clinic was established in 1975 at the Clarke Institute of Psychiatry (now the Centre for Addiction and Mental Health). At the time of this study, the Amsterdam clinic served as the main centre of referral for all gender dysphoric adolescents in the Netherlands. In terms of population size, the Toronto clinic serves a catchment area comparable to the Dutch population and over 90 % of referred clients are from Toronto and its surrounding areas or from other parts of the province of Ontario. Since the year 2000, both clinics have recommended puberty suppression treatment via gonadotropin-releasing hormone agonists (GnRHa) [26] for about two-thirds of the adolescents [27, 28]. However, in the present study, none of the adolescents from both clinics had started this treatment at the time of the baseline assessment.

The Amsterdam sample consisted of 139 adolescents between the ages of 13–18 years referred and assessed between 1996 and 2008. The Toronto sample consisted of 177 adolescents in the same age range referred and assessed between 1980 and 2010. For the demographic characteristics of the sample as a function of clinic, see Table 1. By clinician interview, all adolescents met DSM criteria either for Gender Identity Disorder or Gender Identity Disorder Not Otherwise Specified. None of the adolescents in the current study were seen as child patients in Cohen-Kettenis et al. [20], but their TRF data were used in Steensma et al. [21].

**Table 1:** Demographic characteristics of gender dysphoric adolescents by clinic

	Clinic			
	Amsterdam	Toronto	*t* or *χ* ^2^	*p*
Age (in years)				
*M*	15.69	15.92	1.48	ns
SD	1.46	1.27		
*n*	139	177		
Gender				
Males (%)	79 (56.8)	94 (53.1)	<1	ns
Females (%)	60 (43.2)	83 (46.9)		
M:F ratio	1.31:1	1.13:1		
Full-Scale IQ				
*M*	95.79	97.76	<1	ns
SD	16.45	19.08		
*n*	92	163		
Social class (%)^a^				
I	52 (49.5)	90 (51.1)	<1	ns
II–III	29 (27.6)	49 (27.8)		
IV–V	24 (22.9)	37 (21.0)		
*n*	105	176		
Parent’s marital status (%)^b^				
Both parents	57 (50.0)	77 (43.5)	1.18	ns
Other	57 (50.0)	100 (56.5)		
*n*	114	177		

^a^For parents’ socioeconomic class, see text for classification

^b^For marital status, the category “Other” includes the following family constellations: single parent, separated, divorced, widowed, reconstituted (e.g., mother and stepfather), living in a group home, etc

### Procedure

In both clinics, demographic information and ratings of psychological functioning of the adolescents using the CBCL and the YSR were obtained at the time of assessment. Because in some cases either the parents or the adolescents did not complete the questionnaires, out of the 139 consecutively referred adolescents, CBCL data were available for 112 (80.6 %) adolescents and YSR data were available for 106 (76.3 %) adolescents in the Amsterdam clinic. Because the YSR was introduced to the diagnostic procedure later than the CBCL in the Toronto sample and in some cases either the parents or the adolescents did not complete the questionnaires, out of the 177 consecutively referred adolescents, CBCL data were available for 142 (80.2 %) adolescents and YSR data were available for 138 (78.0 %) adolescents in the Toronto clinic.

### Measures

#### Demographics

The two clinic groups of adolescents were compared on five demographic measures: (1) natal sex of the adolescent; (2) age at assessment; (3) Full-Scale IQ; (4) parents’ marital status; and (5) parents’ social class. We assessed IQ using the American or Dutch versions of the Wechsler Intelligence Scale for Children or the Wechsler Adult Intelligence Scale. Marital status of the parents was categorized as either living with both parents (including adoptive parents from birth) or all other categories (e.g., single parent, separated, divorced, widowed, reconstituted, living in a group home, etc). To estimate parents’ social class, Hollingshead’s [29] Four-Factor Index of Social Status was used, classifying individuals on a 5-point scale ranging from I (major business and professional) to V (Unskilled laborers, menial service workers). The Hollingshead’s ratings were then dummy coded where a social class ranking of I = 1, II–III = 2, and IV–V = 3.

#### Child Behavior Checklist

The CBCL was completed by the parents (over 90 % were mothers) of the adolescents to measure behavioral and emotional problems using the American and Dutch versions in the respective clinics [17, 30]. The CBCL consists of 118 items. Each item was rated on a 3-point scale for the past 6 months: 0 = “not true”, 1 = “somewhat or sometimes true”, and 2 = “very true or often true”. In the Toronto sample, we used maternal ratings for the majority of the adolescents but if the mother was not available, we used ratings by the father or other important adults involved with the adolescent. In the Amsterdam sample, either the parents completed the CBCL together or it was the mother alone. In the Toronto clinic, the CBCL was first used in 1980 and in the Amsterdam clinic it was first used in 1996.

In the present study, four main dependent variables from the CBCL were used: (1) the mean Total Problem score, i.e., the sum of all items rated 1 or 2; (2) the *T* score for Internalizing problems; (3) the *T* score for Externalizing problems; (4) and clinical range scores (>90th percentile) for these three indices. Internalizing and Externalizing *T* scores were calculated using the Dutch norms for the Amsterdam clinic and the American norms for the Toronto clinic, respectively.

On the CBCL, there are two items specifically related to cross-gender behavior: Item 5 (“Behaves like the opposite sex”) and Item 110 (“Wishes to be the other sex). In addition to these items, parents might endorse other items on the CBCL where there is the possibility to give additional remarks that reflect a youth’s cross-gender identification (e.g., Item 85: “Strange ideas,” with a descriptor such as “He thinks he is a girl”). As described in previous studies [19, 24] in order to avoid an artificial inflation in the calculation of behavior problems on the CBCL, we artificially set the value to “0” if Items 5 and 110 were scored as a 1 or a 2 and the same was done for any other item if the parent identified gender-related issues.

#### Youth Self-Report

The YSR, designed for youth between the ages of 11 and 18 years, was administered to the adolescents to measure behavioral and emotional problems using the American and Dutch version in the respective clinic samples [19, 31]. The YSR consists of 102 items (excluding an additional 16 socially desirable “filler” items). Each item was rated on a 3-point scale for the past 6 months with the same verbal anchor points that are used for the CBCL. The dependent measures were identical to the measures used for the CBCL. In the Toronto clinic, the YSR was first used in 1986 and in the Amsterdam clinic it was first used in 1996.

#### Poor peer relations

Following the procedure by Zucker et al. [22], we created a Peer Relations Scale from three CBCL items: “Does not get along with other kids” (Item 25), “Gets teased a lot” (Item 38), and “Not liked by other kids” (Item 48). In Zucker et al. [25], Cronbach’s alpha was .82 for this CBCL scale in an adolescent sample. Likewise, a Peer Relations Scale was constructed from the corresponding YSR items. In Zucker et al. [25], Cronbach’s alpha was .63, which is considered acceptable for research purposes. In that study, the CBCL-YSR correlation for this scale was 45.

### Statistical analyses

We conducted either *t* tests or chi-square tests to compare the demographic variables between the two clinics. For the CBCL and YSR data, parametric statistics (ANOVA or ANCOVA) were used for dimensional measures and non-parametric statistics for dichotomous measures. Effect sizes are reported using Cohen’s *d*. Multiple linear regression was used to identify predictors of behavioral and emotional problems on both the CBCL and YSR.

## Results

### Preliminary analysis: internal validity of the sample

To examine whether or not the included vs. excluded adolescents differed in potentially important ways, we conducted several preliminary analyses of demographic characteristics. The results of chi-square tests revealed no significant differences between the two clinics for the percentage of excluded adolescents on either the CBCL (Amsterdam 19.4 %, Toronto 19.8 %) or the YSR (Amsterdam 23.7 %, Toronto 22.0 %).

We also examined the demographic data for each clinic separately for the five demographic variables of sex, age at assessment, Full-Scale IQ, and parents’ marital status and social class between the included and excluded adolescents. In the Amsterdam sample, we found only one marginally significant difference in demographic measures between the included and excluded individuals. For the YSR, the excluded adolescents had a lower Full-Scale IQ than the included adolescents, *t*(90) = 1.97, *p* = .052. In the Toronto clinic, we found only one marginally significant difference in demographic measures between the included and excluded individuals. For the YSR, the excluded adolescents were younger than the included adolescents with regard to age at assessment, *t*(175) = 1.93, *p* = .056.

### Demographic variables

Table 1 shows the demographic data for each clinic. A *t* test revealed no significant between-clinic differences with regard to age at assessment and Full-Scale IQ. Chi-square analyses revealed no significant differences between the two clinic samples with regard to sex ratio or social class and marital status of the parents.

For the sum of the two CBCL gender items (5 and 110), a 2 (Sex) × 2 (Clinic) analysis of variance (ANOVA) showed a significant main effect for Sex, *F*(1, 253) = 13.21, *p* < .001, *d* = .44. On average, more cross-gender behavior was reported for natal girls than for natal boys. Likewise, for Items 5 and 110 on the YSR, a 2 (Sex) × 2 (Clinic) ANOVA also showed a significant main effect for Sex, *F*(1, 243) = 29.23, *p* < .001, *d* = .68. On average, natal girls self-reported more cross-gender behavior than natal boys.

### Behavioral and emotional problems on the CBCL and on the YSR

Table 2 shows the CBCL and YSR measures as a function of sex and clinic. For the CBCL Total Problem score, a 2 (Sex) × 2 (Clinic) ANOVA revealed a significant main effect for Clinic, *F*(1, 253) = 24.63, *p* < .001, *d* = .64. On average, the Toronto adolescents had more behavioral and emotional problems than Amsterdam adolescents.

**Table 2:** Ratings of behavioral disturbance for the three indices on the Child Behavior Checklist and the Youth Self-Report as a function of sex and clinic

	*n*	Total Problem score^a^		Internalizing *T*		Externalizing *T*		
		*M*	SD	*M*	SD	*M*		SD
CBCL								
Amsterdam	112	45.59	26.75	64.14	10.89		59.48	11.62
Males	63	45.84	26.00	65.11	10.67		58.14	11.94
Females	49	45.27	27.95	62.90	11.17		61.20	11.09
Toronto	142	64.47	31.81	68.78	9.83		62.89	10.82
Males	75	69.27	32.35	69.95	8.99		64.79	11.01
Females	67	59.10	30.55	67.48	10.61		60.76	10.28
YSR								
Amsterdam	106	51.62	24.81	61.53	12.52		54.77	11.72
Males	58	50.16	25.31	63.02	12.95		51.72	11.75
Females	48	53.40	24.33	59.73	11.86		58.46	10.67
Toronto	138	64.07	28.29	62.41	11.96		56.72	10.89
Males	71	66.14	31.17	64.55	13.03		56.49	11.73
Females	67	61.88	24.92	60.15	10.34		56.97	9.99

^a^Absolute range, 0–238 for the CBCL and 0–204 for the YSR

A 2 (Sex) × 2 (Clinic) × 2 (Factor: Internalizing vs. Externalizing) ANOVA yielded a significant Sex × Clinic × Factor interaction, *F*(1, 253) = 7.46, *p* = .007. Post-hoc tests showed that the Toronto boys and girls had significantly higher Internalizing *T* scores than the Amsterdam boys and girls (respective *p*s < .01 and < .03). For the Externalizing *T* score, the Toronto boys had significantly higher scores than the Amsterdam boys (*p* < .001) whereas the Externalizing *T* scores of the Amsterdam and Toronto girls were comparable. Post-hoc tests also showed that both the natal boys and girls from Toronto and the natal boys from Amsterdam had significantly higher Internalizing scores than Externalizing scores (all *p*s < .001), but the two broad-band scores did not differ significantly for the natal girls from Amsterdam.

For the YSR Total Problem score, a 2 (Sex) × 2 (Clinic) ANOVA revealed a significant main effect for Clinic, *F*(1, 243) = 12.36, *p* = .001, *d* = .46. On average, the Toronto adolescents reported more behavioral and emotional problems than Amsterdam adolescents.

A 2 (Sex) × 2 (Clinic) × 2 (Factor: Internalizing vs. Externalizing) ANOVA yielded a significant Sex × Factor interaction, *F*(1, 240) = 28.09, *p* < .001. Post-hoc tests showed that both the natal boys and natal girls had a significantly higher Internalizing score than Externalizing score (*p*s < .001 and < .01, respectively). The natal boys had a significantly higher Internalizing score than did the natal girls (*p* < .02) whereas the natal girls had a significantly higher Externalizing score than did the natal boys (*p* < .03).

#### Clinical range scores

Table 3 shows the percentage of adolescents in each clinic whose CBCL and/or YSR Total Problem score, Internalizing *T* score, and Externalizing *T* score fell in the clinical range (>90th percentile).

**Table 3:** Percentage of adolescents with clinical range scores for the three indices on the Child Behavior Checklist and the Youth Self-Report as a function of sex and clinic

	*n*	Total problem	Internalizing	Externalizing
		Clinical range (%)	Clinical range (%)	Clinical range (%)
CBCL				
Amsterdam	112	55.4	53.6	43.8
Boys	63	57.1	65.1	39.7
Girls	49	53.1	38.8	49.0
Toronto	142	77.5	74.5	48.2
Boys	75	81.3	78.7	56.0
Girls	67	73.1	69.7	39.4
YSR				
Amsterdam	106	40.6	45.3	18.9
Boys	58	41.4	56.9	13.8
Girls	48	39.6	31.2	25.0
Toronto	138	39.9	46.4	25.4
Boys	67	42.3	54.9	22.5
Girls	71	37.3	37.3	28.4
CBCL adolescent norm samples				
Netherlands				
Boys: non-referred	440	9	9	10
Girls: non-referred	456	9	8	8
Boys: referred	328	64	52	59
Girls: referred	254	66	54	47
US				
Boys: non-referred	250	10	10	9
Girls: non-referred	250	10	5	4
Boys: referred	250	71	62	66
Girls: referred	250	74	58	52
YSR adolescent norm samples				
Netherlands				
Boys: non-referred	495	9	9	9
Girls: non-referred	521	8	8	9
Boys: referred	418	31	32	26
Girls: referred	355	38	45	23
US				
Boys: Non-referred	388	9	11	10
Girls: Non-referred	391	12	12	12
Boys: referred	366	30	33	39
Girls: referred	349	40	39	33

Percentages of boys and girls scoring in the clinical range extracted from the Dutch CBCL and YSR manual [30, 33] for the Amsterdam sample and from the American CBCL and YSR manual for the Toronto sample [17, 19]

Between clinics, a significantly greater percentage of adolescents scored in the clinical range in the Toronto clinic compared to the Amsterdam clinic on the CBCL Total Problem score, *χ*^2^(1) = 13.99, *p* < .001, the CBCL Internalizing *T* score, *χ*^2^(1) = 12.02, *p* = .001, but not on the CBCL Externalizing *T* score. There were no significant differences on any of the YSR measures.

A significantly greater percentage of the Toronto girls had a score in the clinical range for the CBCL Total problem score, *χ*^2^(1) = 9.59, *p* = .002, compared to the Amsterdam girls, but not on any of the other five measures. The percentage of boys scoring in the clinical range was significantly higher in the Toronto clinic than in the Amsterdam clinic for the CBCL Total problem score, *χ*^2^(1) = 4.99, *p* = .025, and the CBCL Internalizing *T* score, *χ*^2^(1) = 10.99, *p* = .001, but not for the CBCL Externalizing *T* score or any of the YSR problem scores.

Across both clinics, for the six measures of emotional and behavioral problems, a significantly greater percentage of boys scored in the clinical range compared to girls for the CBCL and YSR Internalizing *T* scores, *χ*^2^(1) = 7.03, *p* = .008 and *χ*^2^(1) = 10.83, *p* = .001, respectively, but not for the CBCL and YSR Total Problem scores and CBCL and YSR Externalizing *T* scores. In the Amsterdam clinic, the percentage of boys scoring in the clinical range was significantly higher than for the girls for the CBCL and YSR Internalizing *T* scores, *χ*^2^(1) = 7.67, *p* = .006, and *χ*^2^(1) = 6.97, *p* = .002, but not for the Total Problem scores and the Externalizing *T* scores. In the Toronto clinic, the percentage of boys scoring in the clinical range was significantly higher than for the girls for the CBCL Externalizing *T* score, *χ*^2^(1) = 3.87, *p* = .049 and for the YSR Internalizing *T* score, *χ*^2^(1) = 4.03, *p* = .038, but not for the other four measures.

### Peer Relations Scale

Table 4 shows the mean score for the Peer Relations Scale as a function of sex and clinic for both the CBCL and the YSR. For this analysis, we covaried the sum of all of the other problems on the CBCL or YSR.

**Table 4:** Ratings on the Peer Relations Scales as a function of clinic and sex

	*n*	Poor peer relations^a^	
		*M*	SD
CBCL			
Amsterdam	112	1.52	1.65
Boys	63	1.83	1.85
Girls	49	1.12	1.29
Toronto	142	2.88	1.88
Boys	75	3.28	1.72
Girls	67	2.43	1.96
YSR			
Amsterdam	106	1.42	1.56
Boys	58	1.69	1.67
Girls	48	1.10	1.37
Toronto	138	2.41	1.78
Boys	71	2.75	1.75
Girls	67	2.06	1.75

^a^Absolute range, 0–6

On the CBCL, a 2 (Sex) × 2 (Clinic) analysis of covariance (ANCOVA) yielded significant main effects for Clinic, *F*(1, 253) = 16.68, *p* < .001, *d* = .76, and Sex, *F*(1, 253) = 11.23, *p* = .001, *d* = .39. On average, adolescents in Toronto had poorer peer relations than those in Amsterdam and boys had poorer peer relations than girls. On the YSR, a 2 (Sex) × 2 (Clinic) ANCOVA yielded significant main effects for Clinic, *F*(1, 243) = 11.50, *p* < .001, *d* = .59, and Sex, *F*(1, 243) = 11.75, *p* = .003, *d* = .35. On average, adolescents in Toronto had poorer peer relations than those in Amsterdam and boys had poorer peer relations than girls.

### Predictors of CBCL and YSR behavioral and emotional problems

A multiple linear regression analysis was conducted for the combined sample as well as separately for boys and girls. The equation was built using direct entry. There were seven independent (predictor) variables: clinic, age, Full-Scale IQ, parents’ social class and parents’ marital status, the sum of the two CBCL/YSR gender items, and the CBCL/YSR Peer Relations Scale. The dependent (criterion) variable was the CBCL/YSR Total Problem score (the sum of CBCL and YSR items rated as a 1 or a 2), without the three items from the Peer Relations Scale.

Table 5 shows the results of the regression analysis. For both the CBCL and the YSR Total Problem score (collapsed across natal sex of the adolescents), the Peer Relations Scale was the strongest predictor. For the CBCL Total Problem score, social class, Full-Scale IQ, and Clinic were also significant predictors. Adolescents with poorer peer relations, from a lower socioeconomic background, with a lower IQ, and from the Toronto clinic showed more behavioral and emotional problems. For boys, social class and Clinic were significant predictors and for girls Full-Scale IQ was also a significant predictor. For the YSR, only Poor peer relations was a significant predictor.

**Table 5:** Predictors of Child Behavior Checklist and Youth Self-Report total behavior problems

Criterion	Significant predictor variables	*B*	*p*
Sum of items (CBCL)	Peer Relations Scale	8.26	<.001
	Social class	2.53	.012
	Full-Scale IQ	−2.66	.008
	Clinic	−2.28	.023
Sum of items (CBCL, boys)	Peer Relations Scale	5.86	<.001
	Social class	3.29	.001
	Clinic	−2.40	.018
Sum of items (CBCL, girls)	Peer Relations Scale	5.77	<.001
	Full-Scale IQ	−2.64	.009
Sum of items (YSR)	Peer Relations Scale	8.09	<.001
Sum of items (YSR, boys)	Peer Relations Scale	6.21	<.001
Sum of items (YSR, girls)	Peer Relations Scale	5.26	<.001

Seven predictor variables were entered into the regression analysis: clinic, age, Full-Scale IQ, parent’s social class, marital status, CBCL gender items (Item 5 and Item 110), and the Peer Relations Scale. Clinic was dummy coded as 1 = Toronto; 2 = Amsterdam. Parent’s marital status was dummy coded from 1 to 3, where 1 = high and 3 = low. Parent’s marital status was dummy coded as 1 = two parents; 2 = other. The criterion variable was the CBCL/YSR Total Problem score (the sum of all items rated 1 or 2, without the three items from the Peer Relations Scale). For CBCL, *n* = 254; for YSR, *n* = 244

## Discussion

The current study is the third in a series of cross-national, cross-clinic comparative analyses of behavioral and emotional problems in gender dysphoric children and adolescents in the Toronto and Amsterdam clinics. When compared to the non-referred CBCL and YSR participants in the standardization samples [17, 19]. The data from the current study showed that the percentage of adolescents with clinical range problems were substantially higher at both sites; however, the percentage with clinical range scores was similar to the referred participants (Table 3). On the CBCL, natal boys from both Toronto and Amsterdam had significantly higher Internalizing than Externalizing *T* scores, as did the natal girls from Toronto. On the YSR, natal boys had more Internalizing than Externalizing problems whereas the reverse was found for girls. With the exception of the natal girls from Toronto on the CBCL, there was a general pattern of an “inversion” of Internalizing vs. Externalizing problems in relation to the sex-typical pattern of more Internalizing problems in girls and more Externalizing problems in boys [32, 33].

Between site comparisons showed that, on both the CBCL and the YSR, the Toronto adolescents had, on average, significantly more behavioral and emotional problems than the Amsterdam adolescents, with moderate effect sizes according to Cohen [34]. The same pattern for CBCL Internalizing and Externalizing *T* scores was found for the boys, with Toronto adolescents having higher scores than the Amsterdam adolescents; the Toronto girls also had significantly higher Internalizing *T* scores than the Amsterdam girls. In our prior studies, we did not find significant CBCL behavior problem differences between the prepubertal children (age 3–12 years) in the two clinics [20]; however, on the TRF, we found that the Toronto children and adolescents had, on average, significantly more behavioral and emotional problems than the Amsterdam adolescents [21].

When we consider the findings from all three studies together, the data appear to indicate that children and adolescents with gender identity problems show elevated rates of behavioral and emotional problems compared to non-referred samples but fairly comparable to clinic-referred samples. Other studies using different methods confirm these findings, although the severity and amount of psychopathology varies, but emotional problems like depression and anxiety are frequently reported [11–14, 16, 24].

When we detected between-clinic differences, the Toronto adolescents always showed more problems than the Amsterdam adolescents. In the TRF study [21], we introduced the argument that one explanation for the greater degree of behavioral and emotional problems in the Toronto adolescents compared to the Amsterdam adolescents is likely due to a greater tolerance or acceptance of gender-variant behavior in Dutch culture than in North American culture. Indeed, in another cross-cultural comparison study between the Netherlands and North America on children growing up in planned lesbian families, there were differences in psychosocial adjustment in favor of the Dutch sample and these appeared to be partly mediated by differences in experienced homophobia [35].

In our previous two studies and in the current one, it does not appear to be the case that demographic differences between the two clinics account for the greater degree of behavioral and emotional problems in the Toronto adolescents, since the two groups were, by and large, comparable on these parameters. It is also very unlikely that any differences in psychopathology between the two clinics is due to any kind of gross sampling bias, in that in both countries there is universal access to health care. The differences are also unlikely to be a function of availability of GnRH agonists for delay or suppression of biological puberty, because the Toronto clinic has adopted the “Dutch protocol” for such treatment [36] shortly after it was introduced in the Amsterdam clinic in the late 1990s [28].

Regarding the measure of poor peer relations, on both the CBCL and the YSR, boys had more problems than girls in both clinics, with a small to moderate effect size, and the Toronto adolescents had more problems than the Amsterdam adolescents, with a moderate to large effect size. In our CBCL study of children [20] and in our TRF study of both children and adolescents [21], we also found that boys had more peer relationship problems than girls and, in the TRF study, we also found that the Toronto children and adolescents had more such problems than the Amsterdam children and adolescents. In the two previous studies, poor peer relations was the strongest predictor of CBCL and TRF behavioral and emotional problems, which was confirmed in the present study as well (Table 5).

These findings are in line with other studies that show social ostracism and peer victimization to be risk factors accounting for co-occurring general psychopathology in gender non-conforming children and adolescents [5–8, 37, 38]. This consistent pattern suggests that one way to reduce co-occurring psychopathology in children and adolescents with gender dysphoria is to improve their standing within peer culture by fostering greater acceptance of variation in gender expression (for a discussion, see Shiffman et al. [39]). Poor peer relations are also partly accounting for the gender differences, with the natal boys apparently experiencing more peer relation problems than the natal girls. As we have suggested before, cross-gender identification may lead to fewer peer relation problems in natal girls compared to natal boys.

However, like studies in the general population on sexual minority stress in LGBT youth, not all variance in psychopathology is accounted for by peer victimization [7]. In the Toronto clinic, for example, it has also been shown that a composite measure of maternal psychopathology predicts variation in general behavioral and emotional problems, suggesting a generic risk factor is at play [40]. This was also evident in the present study, in that we found that Full-Scale IQ (lower) and parent’s social class (lower) were significant predictors of CBCL behavioral and emotional problems. What is also less studied is the role of self-perceived shame and experienced stigma. In one study, there was evidence that emotional dysregulation, which may be a result of chronic stress, mediated the development of emotional problems [5]. The incongruence between one’s experienced/expressed gender and natal sex is likely another source of the distress that gender-dysphoric youth experience.

In conclusion, results of all three studies, using different age groups and informants, showed a similar pattern, with significant behavioral and emotional problems co-occurring in both gender-dysphoric children and adolescents, as reported by the parents, teachers as well as the youth themselves. In all studies, there was a preponderance of Internalizing problems over Externalizing problems. Most significant, the youth from the Dutch clinic showed fewer problem behaviors than the youth from the Toronto clinic. However, regression analyses in all three studies showed that the strongest predictor for behavioral and emotional problems was the peer relation scale.

This finding is in line with studies in the general population on sexual minority youth, in which transgender youth are underrepresented because of low prevalence, which show that peer victimization and social ostracism are important predictors for the development of lower well-being in gender non-conforming youth and this seems true across cultures and nations. This means that clinicians working with this population should be aware of the fact that gender dysphoric adolescents are a vulnerable group. Future studies should also focus on other factors contributing to the mental health problems that gender dysphoric youth may have, in order to develop comprehensive preventive and treatment strategies.
